# Maintenance of pulmonary rehabilitation benefits in patients with COPD: is a structured 5-year follow-up program helpful?

**DOI:** 10.3906/sag-2101-245

**Published:** 2021-07-10

**Authors:** İpek CANDEMIR, Pınar ERGÜN, Mustafa Engin ŞAHİN

**Affiliations:** Department of Chest Disease, Health Science University Atatürk Chest Diseases and Surgery Education and Research Hospital, Ankara, Turkey

**Keywords:** COPD, structured pulmonary rehabilitation programs, exercise capacity, dyspnea

## Abstract

**Background/aim:**

Pulmonary rehabilitation (PR) has proven useful in patients with chronic obstructive pulmonary disease (COPD), but the benefits decrease over time. We evaluated the effects of a structured follow-up program after PR on patient pulmonary function, dyspnea, body composition, exercise capacity, quality of life, psychological status, i-BODE index, hospitalization status, 5-year survival rate. We explored whether this follow-up program could serve as a maintenance program.

**Materials and methods:**

COPD patients who completed PR attended follow-up visits over 5 years. We administered incremental (ISWT), endurance shuttle walk tests (ESWT), measured body (BMI), fat-free mass indices (FFMI), recorded modified Medical Research Council (mMRC), St. George’s respiratory questionnaire (SGRQ), anxiety-depression scores. We also noted the forced expiratory volume in 1 s (FEV1), the forced vital capacity (FVC), the forced midexpiratory flow (FEF25–75), hospitalization, survival rates before, after PR, and in years 1, 2, 3, 5. This was a retrospective observational study.

**Results:**

Thirty-three COPD patients with a mean age of 58 ± 8 years were enrolled. Twenty-seven (82%) were male. The mean FEV1 was 47 ± 16% of the predicted. After PR, the mMRC scale, SGRQ, anxiety, depression scores; i-BODE index; ISWT, ESWT results improved (all p < 0.001), with the improvements persisting through the first year. Patient body composition, pulmonary function did not differ from the baseline over the 5 years (except for a decrease in the FEF25–75 value in year 5; p = 0.003). The hospitalization rate, i-BODE index did not change significantly over the 5 years, the improvements in the ISWT, ESWT outcomes were preserved for 3 years (p = 0.013/0.005, respectively). The quality-of-life, anxiety scores deteriorated in year 1 (both p < 0.001) and year 3 (p = 0.005/0.010, respectively). The dyspnea, depression scores increased progressively over the 5 years.

**Conclusion:**

Structured follow-up programs with visits at 6-month intervals may effectively maintain improvements in COPD. Long-term randomized controlled studies are needed to verify these results.

## 1. Introduction

Pulmonary rehabilitation (PR) effectively improves the quality of life, exercise capacity, dyspnea, anxiety, and depression of patients with chronic obstructive pulmonary disease (COPD) [1–4]. Recently, it was reported that PR was optimal in terms of improving dyspnea, the quality of life, and exercise capacity in patients of all COPD grades [5]. PR seeks to improve both the physical and psychological conditions of patients and promote long-term adherence to health-enhancing behaviors [2]. In several clinical trials, it was found that after 6–12-week PR programs, the benefits were preserved for about 12–18 months in the absence of any specific maintenance [1–4]. However, the benefits of PR decrease over time; maintenance strategies include community and home-based programs. A few studies found that long-term (> 12 months) maintenance programs effectively maintained PR benefits [6–8]. A recent study also found that a PR maintenance program preserved PR efficacy for more than 3 years [9].

No optimal maintenance program type, content, level of supervision, frequency, or duration has been identified. Here, we present the effects of our structured follow-up program (delivered after supervised multidisciplinary PR) on the maintenance of improvements and the hospitalization and survival rates over a 5-year period. We explored whether a structured follow-up program could serve as a useful maintenance program for COPD patients.

## 2. Materials and methods

### 2.1. Study design

We evaluated data on COPD patients who completed PR between March 2007 and December 2010 and attended follow-ups over the following 5 years. This was thus a retrospective, observational real-life study. Written informed consent was routinely obtained prior to PR. The Atatürk Chest Disease and Research Hospital review board approved the study prior to commencement.

### 2.2. Study population

**Figure 1:**
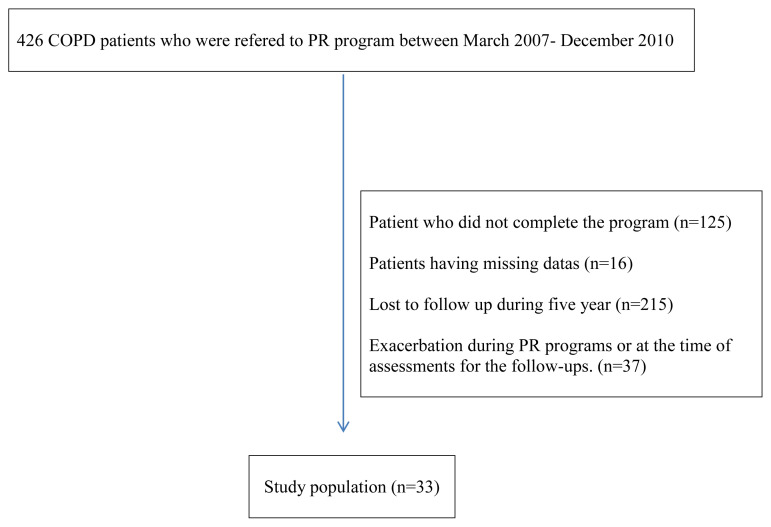
Flow-diagram.

All COPD diagnoses were confirmed by the chest physician of the PR center prior to PR; this is a criterion of the Global Initiative for Chronic Obstructive Lung Disease^1^ [10]. We excluded patients for whom data were missing, those lost to follow-up over the 5 years, and those evidencing exacerbations during PR or follow-up (Figure 1). Patients were grouped by COPD stage based on the postbronchodilator forced expiratory volume in 1 s (FEV1) as follows: stage 1, FEV1/FVC < 70% and FEV1 ≥ 80% of the predicted value; stage 2, FEV1 between 50% and 80% of the predicted value; stage 3, FEV1 between 30% and 50% of the predicted value; and stage 4, FEV1 < 30% of the predicted value^1^ [10].

### 2.3. Outcome parameters

We evaluated the exercise capacity, quality of life, perceived dyspnea, pulmonary function, body composition, and psychological status of the patients before and immediately after the PR program, and in years 1, 2, 3, and 5 of follow-up. Exercise capacity was evaluated using the incremental shuttle walk test (ISWT) and endurance shuttle walk test (ESWT) [10]. Both tests adhered to field walking test guidelines [11]. The minimal clinically important difference (MCID) in the ISWT is 35–36 m [12].

Health-related quality of life was assessed using the St. George’s respiratory questionnaire (SGRQ) [13], and dyspnea was evaluated using the modified Medical Research Council (mMRC) scale [14]. We used spirometry (AS-507 device; Minato Medical Science, Tokyo, Japan) to determine the FEV1, forced vital capacity (FVC), forced midexpiratory flow (FEF25–75), and the FEV1/FVC ratio, in line with American Thoracic Society-European Respiratory Society (ATS-ERS) guidelines [15]. Bioelectrical impedance was applied to assess body composition using a Tanita TBF-300A Total Body Composition analyzer (Tokyo, Japan). The body mass index (BMI) and fat-free mass index (FFMI) were calculated as body mass (for the BMI) and fat-free mass (for the FFMI) in kilograms divided by the square of the height in meters. The Hospital Anxiety and Depression (HAD) scale scores were used to assess psychological status [16].

#### 2.3.1. The i-BODE index

To calculate the validated i-BODE index [17], the 6-min walking distance (6MWD) was replaced by the ISWT distance. It was given in Table 1.

### 2.4. PR program

Patients participated in a hospital-based, outpatient, multidisciplinary, comprehensive PR program on two half-days per week over 8 weeks. The program featured exercise training, education, and nutritional and psychosocial support. Educational sessions hosted by a chest physician, two physiotherapists, a dietician, a nurse, and a psychologist focused on normal pulmonary anatomy and physiology, COPD pathophysiology, communication with healthcare providers, breathing strategies, airway clearance techniques, the roles of medications and why they were prescribed, effective use of respiratory devices, the benefits afforded by exercise and physical activity, energy conservation during daily living, a healthy diet, dietary advice, early recognition and treatment of exacerbations, leisure activities, coping with disease, self-management, and psychosocial issues [2]. Each session ran for approximately 2 h on separate days in a small-group setting at both the beginning and end of PR. Individualized education sessions were scheduled at each follow-up depending on patient needs. Psychological and nutritional support sessions were one-on-one sessions. Psychosocial support sessions focused on coping strategies, cognitive behavior therapy, and stress management [2]. Nutritional intervention sessions included the evaluation of body composition, nutritional status, and nutritional interventions (for example, oral nutritional support) depending on individual daily caloric intake. Patients who were underweight (<21 kg/m^2^), who exhibited unintentional weight loss of >5% over 6 months, and with an FFMI < 17 kg/m^2^ for males or <15 kg/m^2^ for females received oral nutritional support [2,3,18].

Exercise training featured 8 weeks of upper- and lower-limb endurance and resistance training. Endurance training included 30 min of endurance exercise (15 min on a treadmill and 15 min on a stationary bicycle) at 85% of each patient’s VO_2_ peak calculated from the ISWT. Fifteen-minute warm-up and cool-down periods were allowed. Quadriceps resistance training featured leg extensions using free weights on 2 days/week for 8 weeks, with one repetition allowed, commencing at 45% for two sets (10 repetitions/set) and increasing to 70% for three later sets. Resistance training of the shoulder girdle and elbow muscles featured one set of 10 repetitions, commencing at 500 g and progressing to 1–1.5 kg. All training followed guideline recommendations [19,20].

### 2.5. Follow-up

A 5-year follow-up was routine until 2015; the follow-up duration then decreased to 3 years because of the very large number of patients. Follow-up was scheduled at 3-month intervals in year 1 after supervised outpatient PR and every 6 months thereafter. Each follow-up included a physical examination; medical treatment optimization; the ISWT and ESWT; determination of the BMI, FFMI, and mMRC scale and SGRQ scores; pulmonary function testing; and interviews with a psychologist and dietician. Exercise training was represcribed by reference to the PR level at each follow-up. Individual needs and the need for education were evaluated at each follow-up. When a home program was suggested, educational materials including pictures and the schedule of warm-up, cool-down, and breathing exercises as well as walking and resistance training were explained in detail. Endurance training (walking for 30 min) was scheduled based on a target VO_2_ (85% of the individual’s VO_2_ peak calculated using the ISWT). Patients were told to walk more as dyspnea decreased (to Borg ratings of 4–6). When Borg ratings of 4–6 were attained, higher-intensity walking was prescribed (to 85% of the individual’s peak VO_2_ from the ISWT). The resistance training level (using sandbags or bottles filled with water, sand, or beans) depended on the muscle strength evident on follow-up. Patients were told that they could perform one or two extra repetitions over two consecutive days. Patients were encouraged to exercise at least 2–3 days per week. Oral nutritional therapy continued if needed. All PR reassessments were recorded.

### 2.6. Statistical analysis

All statistical analyses were performed with the aid of the Statistical Package for the Social Sciences ver. 18.0 (SPSS, Chicago, IL, USA). A p-value < 0.05 was regarded as indicating significance. Data are presented as the means ± standard deviations or as medians (interquartile ranges). The normality of the data distributions was explored using the Shapiro–Wilk test. Changes in variables with a nonparametric distribution over time (SGRQ anxiety, depression scores, ESWT values) were analyzed using the Friedman test, followed by multiple Wilcoxon signed-rank tests. This yielded p-values which were affected by the number of groups. Changes in normally distributed variables over time (the number of hospitalization, mMRC score, BMI, FFMI, FEV1, FVC, FEF 25–75, ISWT values, i-BODE index) were assessed using a general linear model for repeated measures featuring the Bonferroni adjustment.

## 3. Results

**Figure 2:**
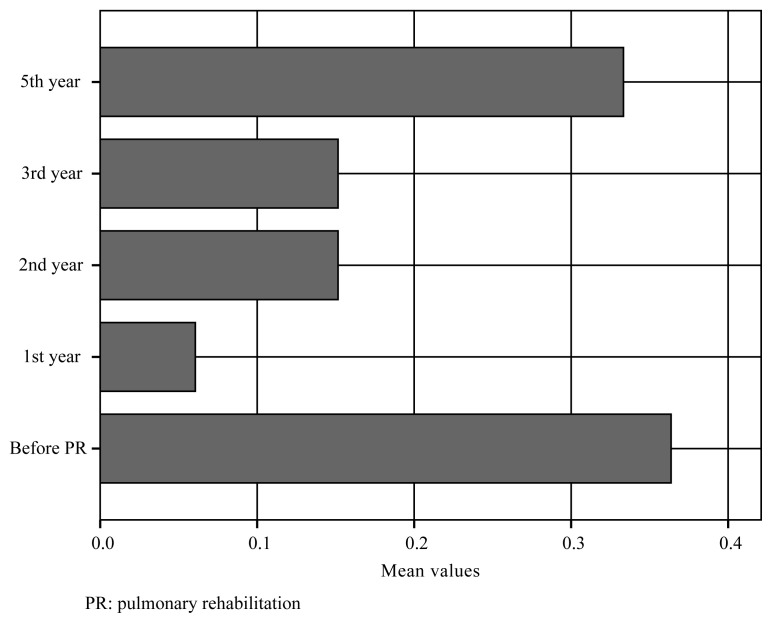
Incremental shuttle walking test (ISWT) values during 5-year period.

**Figure 3:**
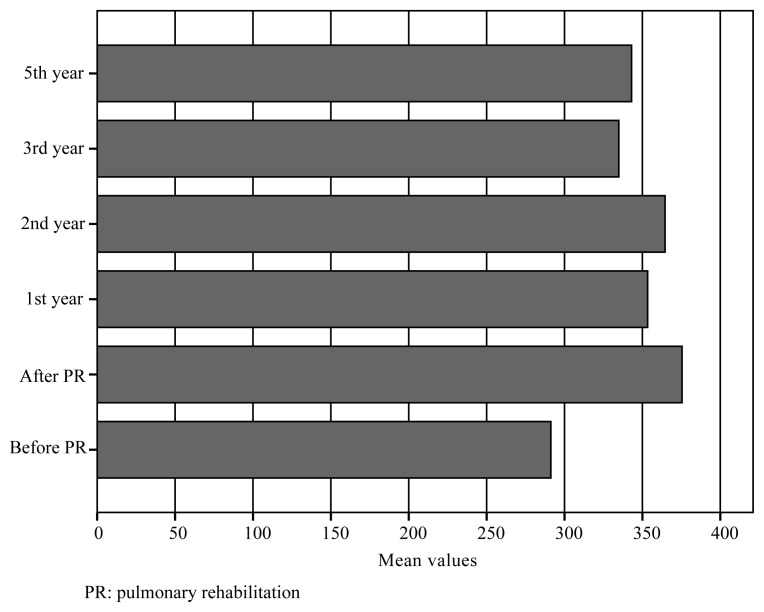
Endurance shuttle walking test (ESWT) values during 5-year period.

We retrospectively enrolled 33 COPD patients with a mean age of 58 ± 8 years, of whom 27 (82%) were male. The mean FEV1 was 47 ± 16% of the predicted value, the mean FVC was 62 ± 17% of the predicted value, and the mean FEV1/FVC ratio was 60 ± 10. Based on spirometry, no patient had stage 1 disease, 14 (42%) had stage 2 disease, 12 (36%) had stage 3 disease, and 7 (21%) had stage 4 disease. Three (9%) patients were current smokers, and 24 (73%) were former smokers [median 30 (60) pack-years]. The mean number of hospitalizations in the year prior to PR was 0.36 ± 0.60. The mean mMRC scale score was 2.5 ± 0.7, the mean SGRQ total score was 52 (30), the mean ISWT distance was 291 ± 100 m, and the median ESWT result was 8 (15) min. After a supervised 8-week PR program, the ISWT (Figure 2) and ESWT (Figure 3) results; mMRC scale, SGRQ, anxiety, and depression scores; and i-BODE index immediately improved (all p < 0.01); however, the FEV1, FVC, BMI, and FFMI did not improve (Table 2).

**Figure 4:**
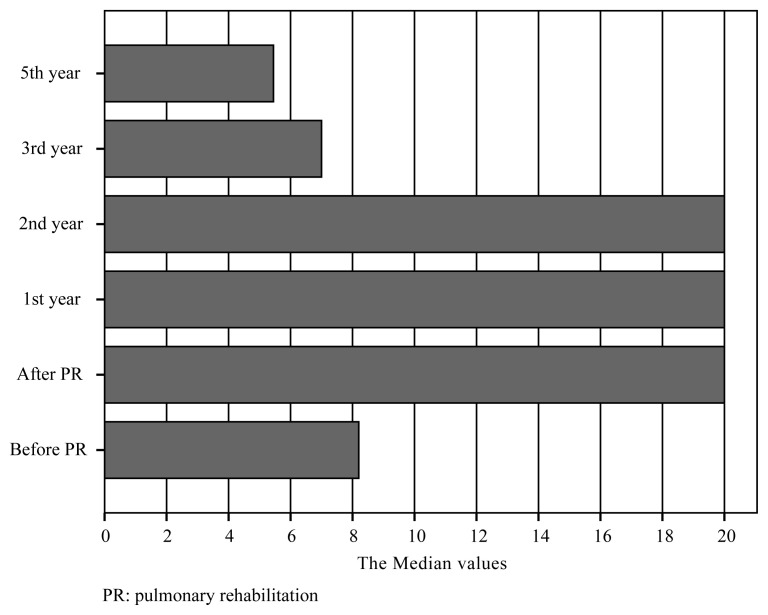
The number of hospitalizations during 5-year period.

We found no differences in the BMI and FFMI after PR or between follow-up visits (p = 0.476, p = 0.141, respectively) over the 5-year period. The mMRC scale scores increased significantly in each of the first 3 years compared to the score at the preceding follow-up (p < 0.001, p = 0.025, p = 0.025, respectively) and exceeded the pre-PR value in year 2. The SGRQ scores increased in years 1 and 3 (p < 0.001, p = 0.005) but only exceeded the pre-PR value in year 5. The ISWT and ESWT results decreased significantly in only year 3 (p = 0.013, p = 0.005, respectively). The ISWT and ESWT results exceeded the pre-PR values in year 5 (Figures 2 and 3). Anxiety increased significantly in year 1 (p < 0.001) and year 3 (p = 0.010), and the anxiety score exceeded the pre-PR score in year 3. The depression score increased in year 1 (p < 0.001), exceeding the pre-PR value, but then did not change significantly (Table 2). The number of hospitalizations decreased significantly in year 1 (p = 0.006) and did not change significantly thereafter (p = 0.32, 0.9, and 0.20 for years 2, 3, and 5, respectively) (Figure 4). The FEV1 and FVC did not change over the 5-year period (FEV1: p = 0.123, 0.512, 0.104, 0.923, and 0.823; FVC: p = 0.066, 0.072, 0.524, 0.312, and 0.120, respectively). The FEF 25–75 values decreased significantly in year 5 (p = 0.003) (Table 2). The i-BODE index increased significantly from 3.9 ± 2.0 to 2.8 ± 1.6 (p < 0.001) and then to 3.5 ± 1.3 (p = 0.010) in year 1 and did not change significantly thereafter. No patient died during the 5-year period.

## 4. Discussion

We found that the dyspnea, exercise capacity, quality of life, anxiety, and depressive symptoms improved in the COPD patients and the number of hospitalizations and i-BODE index decreased after multidisciplinary, comprehensive, supervised hospital-based outpatient PR and that the improvements were maintained over 1 year. Our structured follow-up program (visits at 6-month intervals) may serve as a maintenance program because the decreasing trends in the i-BODE index and the number of hospitalizations persisted for 5 years, and the improvement in exercise capacity persisted for up to 3 years. However, the quality of life and anxiety level deteriorated in both years 1 and 3. The follow-up program did not maintain the PR-induced improvements in dyspnea or depressive symptoms, but it did preserve baseline pulmonary functions and body composition.

Many COPD patients exhibit dyspnea, causing exercise intolerance, which reduces the quality of life, and compromises psychological status. COPD management seeks to reduce symptoms, disease severity, and the number of exacerbations and to improve exercise capacity and health status. This lessens the social and economic burden of disease. PR improves dyspnea, exercise capacity, the quality of life, and psychosocial status; reduces healthcare requirements; and improves the survival of COPD patients whose lung function does not change [[1–4, 21–24], We found that, immediately after PR, dyspnea, exercise capacity, the quality of life, anxiety, depressive symptoms, the number of hospitalizations, and the i-BODE index improved without any change in pulmonary function, which generally declines over time. The FEV1 decline is usually greater in patients with moderate COPD than in those with severe-to-very severe COPD [25, 26]. An annual 15% FEV1 change is clinically meaningful [27]. Regular physical activity slows the decrease in lung function and lessens the risk of COPD development in current smokers [28]. In another study, a 2-year maintenance program following PR prevented an acceleration in FEV1 decline in patients with COPD. In our study, the FEV1 did not decrease significantly (the changes were <15%). This may be attributable to regular exercise, education, the checking of adherence to medications at every follow-up, quit-smoking sessions, and the small number of current smokers. It is thought that the decrease in FEF25–75 over time may reflect a reduction in exercise capacity. A recent study found a strong correlation between the FEF25–75 and exercise capacity [29].

It is important to preserve the benefits of PR long-term. Several strategies have been tested, including telephone contact, monthly supervised sessions, home exercise training [with or without a weekly (supervised) outpatient session], repeat PR, and network programs [9, 29–33]. Our 6-month follow-up program featured multidisciplinary assessments, interviews with a psychologist and dietician, education, and repeated prescription of home exercise training. Although no optimal maintenance program has yet been defined, any such program must consider the structure and resources of PR units/centers. It is not surprising that different models yield different results. One review found that supervised exercise programs after primary PR appeared to be more effective than usual care in terms of preserving exercise capacity for 6 months but not over 1 year. Also, the quality of life did not improve [6]. A cohort study of COPD patients who completed 10 weeks of comprehensive structured home-based PR found that the patients who continued the prescribed exercise at the end of PR maintained their improvements in exercise capacity and psychological and cognitive functioning to the 1-year follow-up [34]. In our study, the gains in exercise capacity, dyspnea, quality of life, and psychological status were preserved in year 1. In a recent study featuring a maintenance network program, improvements in exercise capacity and the quality of life due to PR were preserved for 4 years and the dyspnea benefits for 5 years [9]. In a 3-year follow-up study, after a home exercise program following supervised 8-week PR, the beneficial effects as revealed by the i-BODE index and the 6MWD were maintained for 2 years, but the quality-of-life benefits were not (including the score for the dyspnea domain of the chronic respiratory questionnaire [8]. In our follow-up program with 6-month visits, the improvement in exercise capacity was preserved for up to 3 years. The quality of life deteriorated in years 1 and 3 but was better than baseline up to year 3. The i-BODE index did not change significantly after year 1. This was not unexpected because the three related indices (BMI, FEV1, and exercise capacity) did not change significantly.

The most important long-term goal of PR is survival. Several factors contribute to COPD mortality. Hospitalization caused by COPD exacerbation is one of the most important. Hospital admissions have been found to decrease after PR [35]. The most obvious long-term benefit of our program was reduced hospitalization numbers over the 5 years. Although the baseline hospitalization number was low, 5 years is quite long. Another study found that poor exercise capacity increased mortality; an improvement of at least 30 m in the 6MWD was associated with better outcomes and lower 5-year mortality after PR [36]. In our study, PR-induced improvement in exercise capacity was preserved for up to 3 years; the ISWT distance was 100 m greater than the baseline value immediately after PR and 80 m more in year 5. Both values exceeded the MCID. The repeated prescription of home exercise training at 6-month intervals might have contributed to the high 5-year survival rate. The body composition also predicts mortality; we found that the BMI and FFMI were preserved over the 5 years. The slight decreases in pulmonary function and exercise capacity, together with the preservation of body composition, suggest that a structured, multidisciplinary, supervised, 8-week follow-up PR program decelerates disease progression over 5 years. This is also supported by the preserved i-BODE scores, decreased number of hospitalizations, and high survival rate. Although our results are promising, long-term randomized controlled studies are required.

However, our follow-up program did not preserve the PR-induced improvements in dyspnea and depressive symptoms. Nevertheless, preservation of a better quality of life than that at baseline for up to 3 years is important for COPD patients, more than half of whom had stages 3–4 disease. Also, the increased dyspnea and depression scores may reflect psychological effects rather than the physiological mechanism of dyspnea. Taken together, our results suggest that a structured follow-up program featuring prescribed exercise, as well as comprehensive program reassessment and represcription at each follow-up according to patient needs, contributed significantly to the effective maintenance of PR-induced COPD improvements.

Only a few studies on the long-term maintenance of PR-induced benefits or follow-up PR programs have appeared, especially from countries with few PR centers/units (such as Turkey). As this was a real-life study, our follow-up program is applicable in practice in other PR units. The limitations of our work are that it was a single-center study with a limited number of patients and no control group. Adherence to the home exercise program was not observed. The exclusion of patients lost to follow-up over the 5 years and those who experienced exacerbations during PR or at the times of follow-up would have caused bias, as such patients would likely exhibit higher hospitalization rates and poorer outcomes.

## 5. Conclusion

Comprehensive, hospital-based, supervised, multidisciplinary outpatient PR increased the exercise capacity, quality of life, and psychological status of COPD patients and decreased dyspnea for 1 year. No optimal maintenance program after supervised PR has yet been devised. Randomized controlled studies are needed. However, structured follow-up at 6-month intervals may be effective. Each visit featured comprehensive reassessment and determination of patient needs. This may improve exercise capacity and decrease the number of hospitalizations.

## Figures and Tables

**Table 1:** i – BODE index

Variable	0 points	1 point	2 points	3 points
FEV1 (% predicted)	≥ 65	50–64	36–49	≤ 35
ISWT distance (m)	≥ 250	150–249	80–149	< 80
mMRC scale score	0–1	2	3	4
BMI (kg/m^2^)	> 21	≤ 21		

**Table 2:** The values of parameters.

	Before PR	After PR	p	1st year	p	2nd year	p	3rd year	p	5th year	p
Number of hospitalization	0.36 ± 0.60	-	-	0.06 ± 0.24	**0.006**	0.15 ± 0.45	0.32	0.15 ± 0.50	0.989	0.33 ± 0.90	0.200
BMI (kg/m^2^)	27 ± 6	27 ± 6	0.476	27 ± 6	0.564	27 ± 6	0.665	28 ± 6	0.072	27 ± 5	0.089
FFMI (kg/m^2^)	20 ± 3	20 ± 3	0.141	20 ± 3	0.153	20 ± 3	0.232	20 ± 3	0.432	20 ± 3	0.221
FEV1 % of predicted	47 ± 17	47 ± 17	0.123	46 ± 17	0.512	44 ± 16	0.104	44 ± 16	0.923	44 ± 17	0.823
FVC % of predicted	61 ± 17	61 ± 18	0.066	60 ± 17	0.072	59 ± 18	0.524	59 ± 17	0.312	59 ± 15	0.120
FEF 25–75% of predicted	25 ± 5	25 ± 5	0.426	24 ± 7	0.072	23 ± 7	0.256	23 ± 7	0.672	20+6	**0.003**
SGRQ score	52 (30)	29 (13)	**<0.001**	43 (18)	**<0.001**	40 (19)	0.492	55 (25)	**0.005**	58 (27)	0.304
mMRC score	2.5 ± 0.7	1.9 ± 0.6	**<0.001**	2.4 ± 0.5	**<0.001**	2.7 ± 0.6	**0.025**	3.1 ± 0.6	**0.025**	3.3 ± 0.7	0.800
ISWT (meter)	291 ± 100	375 ± 100	**<0.001**	353 ± 100	0.061	364 ± 120	0.258	334 ± 123	**0.013**	343 ± 127	0.257
ESWT (min)	8 (15)	20 (10)	**<0.001**	20 (15)	0.102	20 (15)	0.879	7 (16)	**0.005**	6 (16)	0.394
Anxiety score	8 (3)	5 (4)	**<0.001**	8 (2)	**<0.001**	8 (4)	0.566	10 (2)	**0.010**	10 (2)	0.564
Depression score	9 (4)	5 (3)	**<0.001**	9 (3)	**<0.001**	10 (3)	0.136	10 (2)	0.149	10 (2)	0.1680
i-BODE index	3.9 ± 2.0	2.8 ± 1.6	**<0.001**	3.5 ± 1.3	**0.010**	3.8 ± 1.3	0.102	4.3 ± 1.6	0.123	4.5 ± 1.6	0.402

BMI: body mass index, FFMI: fat-free mass index, FEV1: forced expiratory volume in 1 s, FVC: forced vital capacity, FEF25–75: forced midexpiratory flow, SGRQ: St. George’s respiratory questionnaire, mMRC: modified Medical Research Council, ISWT: incremental shuttle walking test, ESWT: endurance shuttle walking test, i-BODE index: body-mass index, airflow obstruction, dyspnea, and exercise. *Data were given as mean ± standard deviation and median (interquartile range) according to the normality of data distribution*.

## Footnotes

1 Global Initiative for Chronic Obstructive Lung Disease (2020). Global Strategy for the Diagnosis, Management and prevention of Chronic Obstructive Pulmonary Disease [online]. website: http://goldcopd.org [accessed 10 March 2019].
